# Using machine learning to predict the rupture risk of multiple intracranial aneurysms

**DOI:** 10.3389/fneur.2025.1539341

**Published:** 2025-08-04

**Authors:** Junqiang Feng, Chunyi Wang, Yu Wang, He Liu

**Affiliations:** ^1^Department of Neurosurgery, Beijing Chaoyang Hospital, Capital Medical University, Beijing, China; ^2^Beijing Institute for Brain Disorders, Capital Medical University, Beijing, China

**Keywords:** multiple intra cranial aneurysms, machine learning, risk factors, subarachnoid hemorrhage, risk prediction model

## Abstract

**Objective:**

This study aims to develop a machine learning-based risk prediction model (RPM) for the rupture of multiple intracranial aneurysms (MIAs), addressing a critical gap in current clinical tools such as the PHASES score, which are not specifically designed for MIAs. By analyzing detailed morphological and anatomical parameters, our model provides a tailored approach to rupture risk assessment in MIAs, offering potential improvements over existing methods.

**Methods:**

To address dataset imbalance, we conducted five-fold cross-validation. External validation was not feasible due to data limitations, but we rigorously evaluated model performance using metrics such as accuracy (ACC), true positive rate (TPR), true negative rate (TNR), F1 score, and area under the receiver operating characteristic curve (AUC).

**Results:**

Ninety-one patients with 222 aneurysms were recruited, with a rupture rate of 20.3%. The model demonstrated preferable predication performance in unruptured aneurysms (TNR: 0.837) but showed limitations in predicting ruptured aneurysms (TPR: 0.644). Error analysis revealed that the model’s lower TPR may be attributed to the small sample size and dataset imbalance. Overall, the model achieved an accuracy of 0.797 and an AUC of 0.843.

**Conclusion:**

Our model provides a novel approach to predicting rupture risk in MIAs, complementing existing tools like the PHASES score. However, its clinical applicability is currently limited by suboptimal performance for ruptured aneurysms, which is more suited for identifying MIAs after rupture rather than predicting future rupture risk, and the lack of external validation. Future studies with larger, prospective cohorts are needed to validate and refine the model. This work highlights the potential of machine learning to enhance rupture risk assessment in MIAs, offering a foundation for more personalized treatment strategies.

**Significance:**

Multiple intracranial aneurysms have distinct mechanisms of formation, progression, and rupture. The widely used PHASES score does not incorporate morphological parameters of aneurysms and is not specifically designed for patients with multiple aneurysms. Therefore, we constructed a risk prediction model for the rupture of MIAs by machine learning algorithms.

## 1 Introduction

Multiple intracranial aneurysms (MIAs) refer to the simultaneous presence of two or more intracranial aneurysms (IAs), accounting for approximately 26–30% of all IAs (1, 2). Their rupture risk and treatment strategies are challenging issues in the field of neurosurgery. Rupture of IAs can lead to severe subarachnoid hemorrhage (SAH) with high rates of disability and mortality, representing 5% of all strokes (3–5). Furthermore, the rate of SAH in MIAs is higher than that in single aneurysms (6). Additionally, the rate of surgical complications for MIAs is significantly higher than that for single aneurysms (7). Besides, surgical intervention, such as craniotomy and clipping surgery, imposes risks; thus, not all unruptured MIAs need treatment. Based on these reasons, a careful assessment of the risks and benefits is necessary before surgery (8). Predicting high-risk MIAs and identifying the arteries responsible for SAH can help clinicians make timely treatment decisions and prevent catastrophic outcomes. The PHASES score developed a risk prediction chart to estimate the 5-year rupture risk of unruptured intracranial aneurysms based on the status of specific risk factors. The PHASES score is of significant importance and widely used for risk assessment of unruptured aneurysms. However, morphological parameters of the aneurysms were not included in PHASES score and this score did not specifically focus on MIAs (2). To clarify the correlation between MIAs and the rupture risk, we analyzed data from patients with MIAs in our medical center, including those with or without SAH. This study aimed to explore the relationship between the rupture of MIAs and their anatomical structure and morphological characteristics.

In the past few years, machine learning has been increasingly used by researchers (9, 10). It utilizes clinical data to learn the patterns and structure of a dataset. Some of the previous studies have explored the feasibility of machine learning in cerebral aneurysms (11). In this study, we applied machine learning to construct a risk prediction model (RPM) for the rupture risk of MIAs, to provide a more individualized risk assessment. Then, we validated the accuracy of the model, thereby developing more scientific and precise treatment plans for patients to improve their prognosis and quality of life.

## 2 Materials and methods

### 2.1 Patients and data collection

From October 2012 to November 2022, we retrospectively included 91 patients with MIAs admitted to the Department of Neurosurgery at Beijing Chaoyang Hospital, Capital Medical University. All patients underwent digital subtraction angiography (DSA), performed by experienced neurointerventionists, for confirmation. To ensure consistency, all measurements were performed by at least two trained physicians (with 3 and 8 years of experience, respectively) and using professional measuring tools. Among them, there were 61 female patients (67.0%) with a mean age of 59.2 ± 12.0 years. Data were collected on age, sex, aneurysm anatomical parameters, aneurysm site, shape, and rupture status.

### 2.2 Confirmation of ruptured aneurysms

In this study, the diagnosis of SAH was supposed through clinical presentation and head CT. A 64-slice multidetector GE revolution frontier CT scanner (GE Healthcare, Waukesha, Wisconsin, USA) was used for all patients with suspected SAH. Patients diagnosed with SAH underwent CT angiography (CTA) for three-dimensional reconstruction to confirm the presence of IAs and determine the size and location of the aneurysm. If the presence of aneurysms was suspected in CTA and more information was needed to guide treatment, DSA was performed to clarify the anatomical characteristics of the aneurysm. Patients with initial negative DSA underwent a second DSA examination 1 week later. Aneurysms were measured using two-dimensional images obtained from the Philips FD20 workstation. We used “quantitative automatic calibration” and electronic calipers. The maximum values of anatomical data were measured from at least four projections. We determined the ruptured aneurysms based on the SAH location, combined with the size and shape of the aneurysm.

### 2.3 Inclusion and exclusion criteria

Inclusion criteria were as follows: (1) confirmed IAs based on CTA or DSA, with a minimum of 2 aneurysms; (2) confirmation of SAH by head CT or lumbar puncture; and (3) complete clinical data.

Exclusion criteria were as follows: (1) patients without CTA or DSA results; (2) traumatic, infectious, or dissecting aneurysms; (3) presence of other cerebrovascular diseases that may interfere with the results, such as moyamoya disease, vasculitis, etc.; and (4) incomplete clinical data or poor image quality.

### 2.4 Characteristics and parameters of aneurysm

Morphological characteristics of aneurysms mainly refer to regular or irregular shapes (with protrusions, blebs, or multilobulated appearance) (12).

Aneurysmal parameters included maximum diameter, height, neck width, parent artery diameter, aspect ratio (AR), neck-to-parent artery diameter (NPR), and size ratio (SR), which were standardized to minimize variability. The maximum diameter (L) was the longest distance from the midpoint of the aneurysm neck to the aneurysm dome. The height (H) was the maximum vertical distance from the dome to the plane of the neck. The neck width (D) was the average diameter where the aneurysm joined the parent artery. The parent artery diameter (Dm) was the average diameter of the parent artery and its branches. The average diameter of each branch vessel (Dv) was calculated as the average diameter at the proximal neck (D1) and 1.5 times the diameter D2 at a distance of D1 from the neck. AR is the H/D ratio. NPR is the D/Dm ratio. SR is the L/Dm ratio (13). All measurements were performed by at least two independent and experienced neurointerventionists, and discrepancies were resolved through consensus.

MIAs are mainly distributed in the anterior circulation of the internal carotid artery system and the vertebrobasilar artery system. The anterior circulation of the internal carotid artery system includes the internal carotid artery (ICA), ophthalmic artery, anterior choroidal artery, posterior communicating artery (PcomA), middle cerebral artery (MCA), anterior cerebral artery (ACA), and anterior communicating artery (AcomA). The vertebrobasilar artery system in the posterior circulation includes the basilar artery (BA), posterior cerebral artery (PCA), vertebral artery (VA), etc.

Bifurcation-type aneurysms are defined as aneurysms located at parent artery bifurcations in the circle of Willis that originate from more than one parent vessel (ICA terminus, MCA bifurcation, AcomA, and apex of the BA). Aneurysms originating from only one parent vessel are defined as sidewall aneurysms (14, 15).

Mirror aneurysms refer to aneurysms that symmetrically exist on both sides of the same location in the cranium (16).

Tandem aneurysms refer to two or more aneurysms in adjacent positions on the same parent artery (17).

### 2.5 Model construction and performance evaluation

Given the relatively small sample size in this study and the advantages of the Categorical Features and Gradient Boosting (CatBoost) algorithm in directly handling categorical features, resistance to overfitting, and automated and efficient modeling, this research employed the CatBoost algorithm on the Python platform to construct a predictive model. Additionally, the Optuna hyperparameter optimizer was utilized, with hyperparameter tuning accomplished through the Bayesian optimization algorithm using TPE (Tree-structured Parzen Estimator) (18).

To measure the performance of the model, we calculated accuracy (ACC), true positive rate (TPR), true negative rate (TNR), and the F1-score and Matthew’s correlation coefficient (MCC). In the following formulas, TP, FP, TN, and FN represent the number of true positive, false positive, true negative, and false negative samples, respectively.


$$\mathrm{ACC}=\frac{\mathrm{TP}+\mathrm{TN}}{\mathrm{TN}+\mathrm{FP}+\mathrm{FN}+\mathrm{TP}}$$

$$\mathrm{TRP}=\frac{\mathrm{TP}}{\mathrm{FP}+\mathrm{FN}}$$

$$\mathrm{TNR}=\frac{\mathrm{TN}}{\mathrm{TN}+\mathrm{FP}}$$

$$F1\;\text{score}=\frac{\mathrm{TP}\times 2}{\mathrm{TP}\times 2+\mathrm{FP}+\mathrm{FN}}$$

$$\mathrm{MCC}=\frac{\mathrm{TP}\times \mathrm{TN}-\mathrm{FP}\times \mathrm{FN}}{\sqrt{\left(\mathrm{TP}+\mathrm{FP})(\mathrm{TP}+\mathrm{FN})(\mathrm{TN}+\mathrm{FP})(\mathrm{TN}+\mathrm{FN}\right)}}$$

Moreover, the area under receiver operating characteristic (ROC) curve (AUC) and precision-recall curve (PRC) were used to evaluate the overall predictive performance of the model. The selection of performance metrics was guided by their clinical applicability in optimizing aneurysm rupture detection. We prioritized the F1 score and AUC as primary evaluation criteria, with particular emphasis on their respective capacities to address critical clinical requirements. The F1 score’s harmonic balance between precision (positive predictive value) and recall (sensitivity) directly addresses the dual clinical imperative of minimizing missed ruptures while reducing unnecessary interventions triggered by false positives. This dual consideration is particularly crucial given the catastrophic consequences of undetected ruptures and the iatrogenic risks associated with invasive procedures for false-positive cases. In contrast, the AUC provides a threshold-agnostic evaluation of the model’s discriminative capacity across the entire spectrum of diagnostic decision points. This characteristic makes it particularly valuable for assessing the model’s robustness in clinical scenarios where optimal classification thresholds may vary depending on patient-specific factors or evolving clinical protocols. Additionally, we utilized a class weighting strategy to address the scarcity of positive samples and imbalance between positive and negative samples, and L2 regularization to mitigate the issue of multicollinearity.

### 2.6 Model interpretation

We used a game-theoretic approach named Shapely additive explanations (SHAPE) to explore how the features affect the output of the RPM (19, 20). SHAPE connects optimal credit allocation to local explanations using the classic Shapley values (SHAP values). These values use the ‘Shapley interaction index’ from the game theory to capture local interaction effects from game theory and their extensions. The SHAP value breaks a prediction value into contributions from each feature. It measures the effect of a feature on a single prediction value compared to the baseline prediction (19). In our case, the positive SHAP value made more positive prediction results, while negative SHAP values made more negative prediction results. A positive effect means that the model prefers these feature values in predicting the rupture risk of MIAs. The application of SHAP summary plots and SHAP dependence plots enhances our capacity to effectively interpret and visualize feature impact.

### 2.7 Statistical analysis

All statistical analyses were performed using IBM SPSS Statistics for Windows, version 26 (IBM Corp., Armonk, New York, USA). Continuous variables are presented as mean ± SD, and categorical variables are presented as percentages. Student’s *t*-test and Mann–Whitney *U* test were used to compare continuous variables, and the chi-square test was used to compare categorical variables. Variance inflation factor (VIF) was used to assess multicollinearity among the risk factors. Additionally, logistic regression analyses were performed to determine the predictors of IA rupture in patients with MIAs. *p*-values below 0.05 were considered statistically significant.

## 3 Results

### 3.1 Summary of clinical characteristics

Overall, data from 91 patients and 222 aneurysms were analyzed. There were 61 females (67.0%) and 30 males (33.0%). Their age ranged from 21 to 87 years with a median age of 59.2 ± 12.0 years. Sixty patients (65.9%) had a history of hypertension, 42 patients (46.1%) experienced SAH, and 49 patients (53.8%) did not experience rupture. Detailed data on the characteristics of patients and aneurysms are shown in Table 1. Additionally, patients were divided into ruptured (*n* = 42) and unruptured (*n* = 49) groups based on the presence of aneurysmal SAH. There were no significant differences (*p* > 0.05) in sex, age, history of hypertension, and number of aneurysms between the two groups.

**Table 1:** Patient and aneurysm characteristics.

Patient and aneurysm characteristics	Number (%)
Patient number	91
Aneurysm number	222
Sex (female/male)	61/30 (67.0%/31.0%)
Median age (y) ± SD	59.2 ± 12.0
History of hypertension	60 (65.9%)
History of SAH	42 (46.1%)
Aneurysm size (mm)	4.94 ± 3.13
Morphology	
Regular	153 (68.9%)
Irregular	69 (31.1%)

### 3.2 Anatomical characteristics of MIAs

The median number of aneurysms in each patient was 2.0 (2.0, 3.0), ranging from 2 to 6 aneurysms per patient. Specifically, there were 62 patients (68.1%) with 2 aneurysms, 20 patients (20.0%) with 3 aneurysms, eight patients (8.8%) with 4 aneurysms, and 1 patient (1.1%) with 6 aneurysms (Table 2).

**Table 2:** Number of aneurysms.

Aneurysm frequency	Number
2	62 (68.1%)
3	20 (20.0%)
4	8 (8.8%)
6	1 (1.1%)

Among patients who experienced SAH, there were 45 responsible aneurysms. The ruptured aneurysms had a maximum diameter of 4.86 (3.48, 6.83) mm, a height of 4.00 (2.75, 6.26) mm, a neck width of 3.00 (2.15, 4.03) mm, an AR of 1.35 (1.00, 1.64), an NPR of 1.15 (0.78, 1.54), an SR of 1.33 (0.96, 2.46), and a parent artery diameter of 2.76 (2.05, 3.58) mm. In total, 35 (77.8%) ruptured aneurysms were irregular. As for the largest aneurysms, the maximum diameter was 5.00 (3.90, 7.35) mm, the height was 4.30 (3.00, 6.92) mm, the neck width was 3.19 (2.50, 4.09) mm, the AR was 1.35 (1.00, 1.53), the NPR was 1.17 (0.81, 1.83), the SR was 1.31 (1.04, 2.42), and the parent artery diameter was 3.20 (2.18, 3.73) mm. There were 26 irregular aneurysms (61.9%) (Table 3). Besides, unruptured aneurysms in these patients had a maximum diameter of 4.58 (1.73, 7.43) mm, a height of 3.82 (1.46, 6.81) mm, a neck width of 3.16 (0.94, 5.38) mm, an AR of 1.30 (0.62, 1.98), an NPR of 1.08 (0.46, 1.70), an SR of 1.34 (0.44, 2.24), and a parent artery diameter of 3.17 (1.92, 4.42) mm. Eight (14.5%) unruptured aneurysms were irregular (Table 4).

**Table 3:** Comparison of aneurysmal parameters.

Aneurysmal parameters	Ruptured aneurysms	Largest one of ruptured aneurysms	Largest one of unruptured aneurysms
Maximum diameter (mm)	4.86 (3.48, 6.83)	5.00 (3.90, 7.35)	7.50 (4.75, 9.00)
Height (mm)	4.00 (2.75, 6.26)	4.30 (3.00, 6.92)	5.90 (3.47, 7.90)
Neck width (mm)	3.00 (2.15, 4.03)	3.19 (2.50, 4.09)	4.30 (3.13, 5.65)
AR	1.35 (1.00, 1.64)	1.35 (1.00, 1.53)	1.24 (0.95, 1.67)
NPR	1.15 (0.78, 1.54)	1.17 (0.81, 1.83)	1.05 (0.82, 1.65)
SR	1.33 (0.96, 2.46)	1.31 (1.04, 2.42)	1.56 (1.00, 2.20)
Parent artery diameter (mm)	2.76 (2.05, 3.58)	3.20 (2.18, 3.73)	3.59 (3.04, 4.50)

**Table 4:** Comparison of ruptured and unruptured aneurysm in SAH patients.

Parameter category	Specific metric	Ruptured aneurysms	Unruptured aneurysms
Aneurysmal parameters	Maximum diameter (mm)	4.86 (3.48, 6.83)	4.58 (1.73, 7.43)
	Height (mm)	4.00 (2.75, 6.26)	3.82 (1.46, 6.81)
	Neck width (mm)	3.00 (2.15, 4.03)	3.16 (0.94, 5.38)
	AR	1.35 (1.00, 1.64)	1.30 (0.62, 1.98)
	NPR	1.15 (0.78, 1.54)	1.08 (0.46, 1.70)
	SR	1.33 (0.96, 2.46)	1.34 (0.44, 2.24)
	Parent artery diameter (mm)	2.76 (2.05, 3.58)	3.17 (1.92, 4.42)
Morphology	Regular	10 (22.2%)	47 (85.5%)
	Irregular	35 (77.8%)	8 (14.5%)

Among the 49 patients with 122 aneurysms who did not experience SAH, the maximum diameter of the largest aneurysm was 7.50 (4.75, 9.00) mm, its height was 5.90 (3.47, 7.90) mm, its neck width was 4.30 (3.13, 5.65) mm, its AR was 1.24 (0.95, 1.67), its NPR was 1.05 (0.82, 1.65), its SR was 1.56 (1.00, 2.20), and its parent artery diameter was 3.59 (3.04, 4.50) mm. There were 14 irregular aneurysms (28.6%) (Table 3).

### 3.3 Location of MIAs

The locations of aneurysms are summarized in Table 4. Among the 45 ruptured aneurysms, 29 (64.4%) were located at parent artery bifurcations, including 9 located at AcomA (31.0%) and 7 located at PcomA (24.1%). Of them, 42 (93.3%) originated from the anterior circulation, and 3 (6.67%) originated from the posterior circulation (Table 5).

**Table 5:** Location distribution of aneurysms.

Classification category	Location	Number (%)
Anatomical location	ICA	107 (48.2%)
	MCA	32 (14.4%)
	PcomA	30 (13.5%)
	Posterior circulation	24 (10.8%)
	AcomA	21 (9.5%)
	ACA	8 (3.6%)
Relationship with the parent artery	Bifurcation	77 (34.7%)
	Sidewall	145 (65.3%)

Out of 42 unruptured aneurysms with the largest sizes, 12 (24.5%) were located at parent artery bifurcation, 45 (91.8%) originated from the anterior circulation, and 4 (8.16%) originated from the posterior circulation (Table 5).

### 3.4 Special types of MIAs

Mirror aneurysms consisted of 26 pairs (52 aneurysms), among which 9 aneurysms (17.3%) were ruptured. Among the ruptured ones, 6 (66.7%) were on the right side and 3 (33.3%) were on the left side. There was no statistically significant difference (*p* > 0.05) between the two sides.

Tandem aneurysms included 32 sets (70 aneurysms), among which 8 (11.4%) aneurysms were ruptured. Among them, 6 aneurysms were located at the distal end (75.0%), and 2 were located at the proximal end (25.0%) and no significant difference (*p* > 0.05) was observed in rupture risk between the two groups.

### 3.5 Comparison of aneurysm characteristics between the ruptured and unruptured groups

Statistically significant differences (*p* < 0.05) were found in several characteristics of aneurysms between the ruptured and unruptured groups. For instance, the number of aneurysms in the AcomA and ICA, aneurysms located at parent artery bifurcations, and irregularly shaped aneurysms, maximum diameter, height, neck width, and parent artery diameter were significantly different between the two groups. There were no significant differences in other variables between the two groups. Besides, to assess multicollinearity, which might affect the reliability of regression coefficients in our model, we calculated VIFs of maximum diameter, AR and parent artery diameter and the results were 1.289, 1.267 and 1.050, respectively. The results demonstrate that there is no obvious multicollinearity.

### 3.6 Predicting the rupture risk of MIAs using the RPM

In this section, we showcase the application of the RPM for predicting the rupture risk of MIAs. Five-fold cross-validation experiments were applied to the MIAs datasets and showed that our model had a satisfactory accuracy, with an ACC of 0.797. The model performed well in the prediction of unruptured MIAs, with a TNR of 0.837, but performed relatively weakly in the prediction of ruptured MIAs, with a TPR of 0.644, indicating suboptimal performance for this critical class. For example, misclassified ruptured aneurysms tended to have smaller maximum diameters and lower AR values compared to correctly classified cases. Besides, the model had an F1 score, MCC, AUC, and PRC of 0.567, 0.445, 0.843, and 0.562, respectively (Table 6).

**Table 6:** Comparison of location characteristics.

Classification feature type	Location	Ruptured aneurysms	Largest one of unruptured aneurysms
Anatomical location	ICA	23 (51.1%)	36 (73.5%)
	MCA	6 (13.3%)	6 (12.2%)
	PcomA	0 (0.0%)	0 (0.0%)
	Posterior circulation	3 (6.7%)	4 (8.2%)
	AcomA	10 (22.2%)	2 (4.1%)
	ACA	3 (6.7%)	1 (2.0%)
Relationship with the parent artery	Bifurcation	29 (64.4%)	12 (28.6%)
	Sidewall	16 (35.6%)	30 (71.4%)

**Figure 1:**
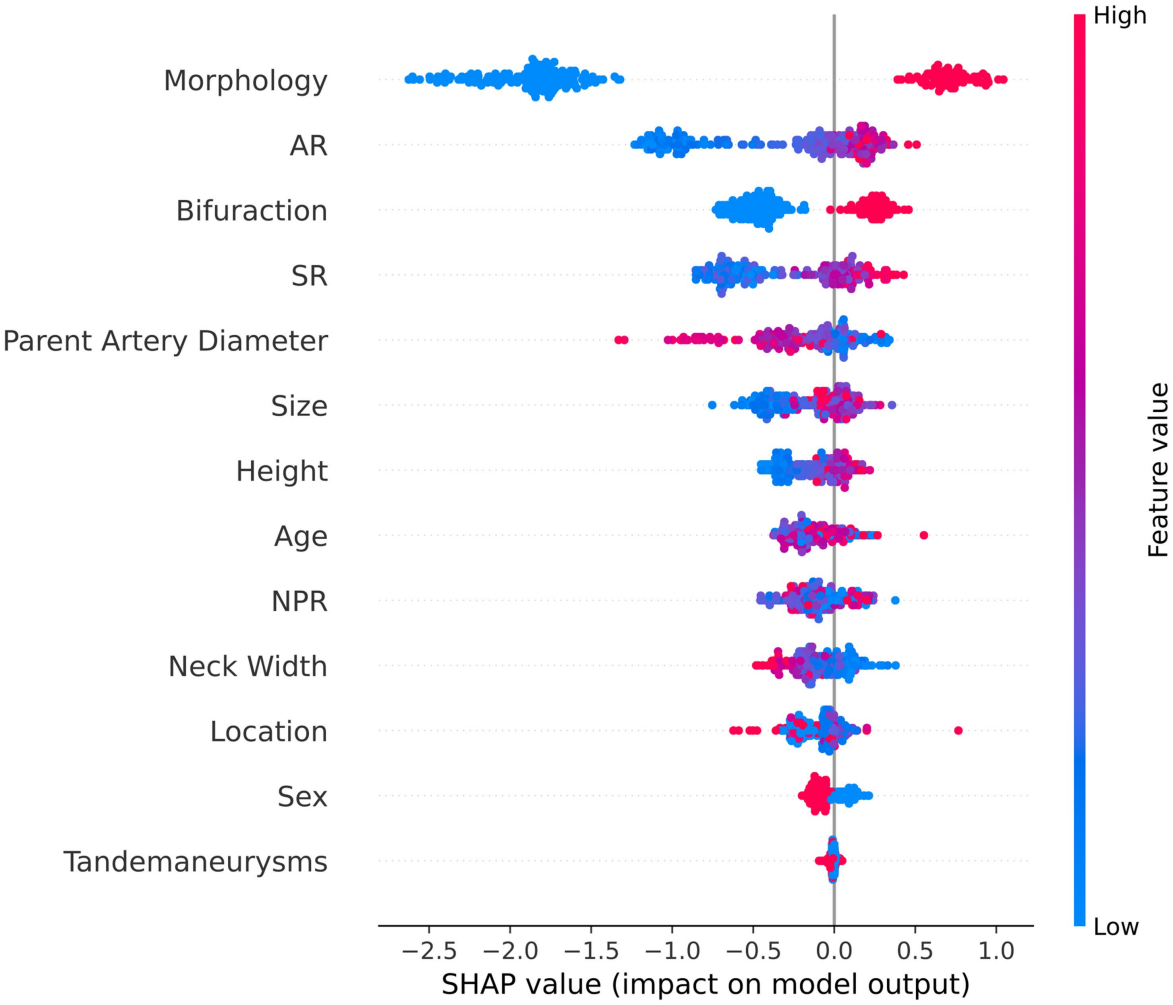
Summary plot of the SHAP values. This figure is a set of beeswarm plots, where each dot corresponds to a sample in the study. The dot’s position on the x-axis shows the effect of each feature on the model’s prediction for that person. When multiple dots land at the same x position, they pile up to show density. The y-axis shows the 13 risk factors associated with MIA rupture.

**Figure 2:**
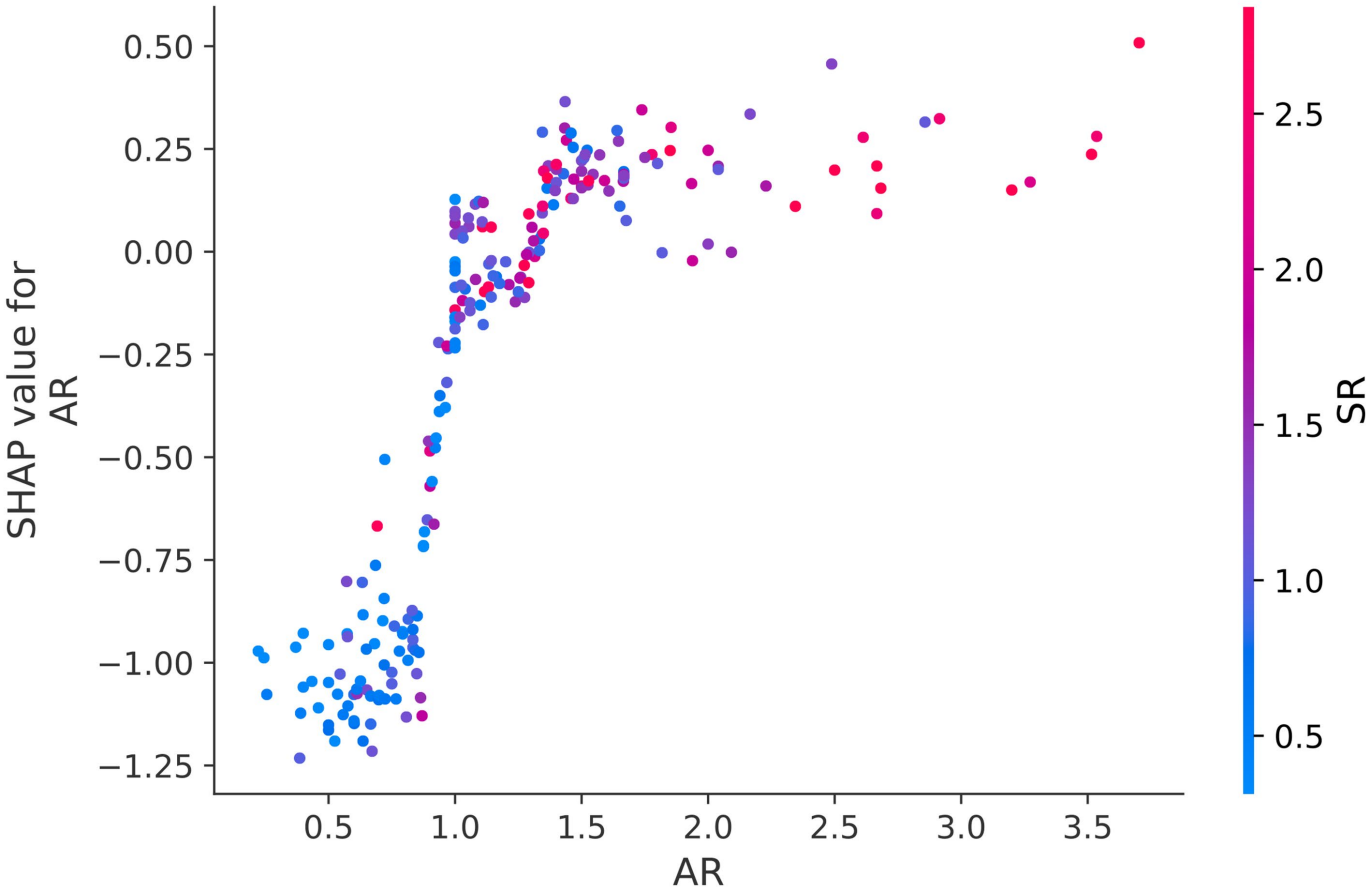
Dependence plot of the interaction of AR and SR. In the above figure, a clear interaction effect is visible between AR and SR, which means that increasing in AR and SR can contribute to the possibility of MIA rupture.

Figure 1 depicts the SHAP summary plot of RPM trained using the MIAs data from patients admitted to our hospital from October 2012 to November 2022. It ranked 13 features closely related to the rupture of MIAs. According to this SHAP summary plot, morphology, AR, bifurcation, SR, and height were important features dramatically affecting the prediction ability of the model (Figure 1). Besides, we detected some features that were highly associated with each other. For instance, the interaction of AR and SR was especially obvious, and these two features positively contributed to model prediction (Figure 2).

Altogether, these results indicated that incorporating an ensemble learning algorithm confers flexibility for predicting the rupture risk of MIAs (Table 7).

**Table 7:** Relevant parameters to evaluate model performance.

Parameter	Number
ACC	0.797
TPR	0.644
TNR	0.837
MCC	0.445
F1 score	0.567
AUC	0.843
PRC	0.562

## 4 Discussion

In this study, we collected data on patients with MIAs with or without SAH to identify the risk factors of aneurysm rupture and demonstrates the potential of machine learning to improve rupture risk prediction in MIAs. Compared to the PHASES score, our RPM offers a more tailored approach to MIAs, leveraging detailed morphological and anatomical parameters. However, the model’s lower TPR for ruptured aneurysms highlights the need for further refinement.

Univariate analysis indicated that there were significant differences between ruptured and unruptured aneurysms in terms of being located in the AcomA and ICA, suggesting a potential association with rupture risk. Detmer et al. (22) suggested that aneurysms originating from MCA and PcomA are at a higher risk of rupture; whereas Silva et al. (21) reported a higher risk of rupture among aneurysms originating from the cavernous segment of the ICA, AcomA, and PcomA. In which location aneurysms are more susceptible to rupture is still unclear, but many studies suggested that aneurysms in the AcomA and PcomA may be at a higher risk of rupture (7, 23, 24). This finding aligns with the general conclusion of this study. In addition, the majority of ruptured aneurysms (64.4%) were located at parent artery bifurcations, particularly in the AcomA (31.0%), PcomA (24.1%), and the apex of BA (6.9%). Other studies have also confirmed that aneurysms located at parent artery bifurcations are at a higher risk of rupture (25, 26). Aneurysms at parent artery bifurcations and sidewalls have different hemodynamic characteristics, which may increase their risk of rupture. Recently, Shimizu et al. (27) found a potential relationship between bifurcation angles and aneurysm growth (28).

Furthermore, in our dataset, the rupture risk of irregular aneurysms was markedly higher than regular aneurysms. Both univariate analysis and logistic regression analysis confirmed that irregular shape can be considered an important risk factor for the rupture of MIAs. Recent studies have demonstrated the association between irregular shape and aneurysm rupture risk (24, 29). Additionally, in the study sample, the average maximum diameter of irregular aneurysms was larger than regular aneurysms. A retrospective study by Qi et al. (30) suggested that the rupture risk of irregular aneurysms is likely related to the size of aneurysms. Using computational fluid dynamics simulations, Cebral et al. (29) found that localized high wall shear stress (WSS) caused by abnormal blood flow can lead to focal wall damage and facilitate the formation of irregular aneurysms.

Our logistic regression analysis revealed a significant difference in AR between ruptured and unruptured aneurysms, with a median AR of 1.35 (1.00, 1.64) for ruptured aneurysms and 1.24 (0.95, 1.67) for unruptured aneurysms. A larger AR may be a risk factor for the rupture of MIAs and AR is regarded as a valid and useful clinical measure to predict aneurysm rupture (31). Backes et al. (14) found that AR more than 1.3 is associated with the risk of aneurysm rupture independent of aneurysm size and location. Additionally, studies showed that high aspect ratio values can lead to lower WSS, and low WSS can induce a chain of atherosclerotic changes and inflammatory reactions, which drive the remodeling of aneurysm wall, aneurysm growth, and rupture (32, 33). Therefore, clinicians should pay attention to unruptured aneurysms with higher AR values. Figure 2 illustrates a clear interaction of AR and SR. They had a combined effect on the prediction model. AR provides a relationship between aneurysm size and aneurysm neck width, while SR indicates the ratio of aneurysm body length to the average diameter of the parent artery (13). Farnoush et al. (34) investigated the relationship between these two morphological parameters and their potential to explain the evolution of bifurcation-type cerebral aneurysms. Their results showed that AR and SR can provide useful information regarding the growth of bifurcation aneurysms, which is related to the hemodynamic parameter of energy loss. Moreover, the SHAP summary plot depicted that the morphology, AR, bifurcation, SR, and height of MIAs can markedly affect the prediction ability of the model (Figure 1). Statistical analyses demonstrated that irregular morphology, being located in the arterial bifurcation, and larger AR are risk factors for aneurysmal rupture. Neurosurgeons should pay attention to these features when choosing surgical or conservative treatments for MIAs.

The PHASES score developed a risk prediction chart to estimate the 5-year rupture risk of unruptured intracranial aneurysms based on the status of specific risk factors. In contrast, this study utilized retrospective data to develop a model that assists clinicians in identifying the ruptured aneurysm among patients with multiple intracranial aneurysms. This study targets patients with multiple intracranial aneurysms, including those with or without ruptured aneurysms, whereas the PHASES score is specifically designed for patients with unruptured intracranial aneurysms. The model presented in this article incorporates risk factors such as morphology, AR, bifurcation, SR, and height. The PHASES score includes factors such as Population, Hypertension, Age, Size of aneurysm, Earlier SAH from another aneurysm, and Site of aneurysm (35). Besides, the presence of multiple coexisting aneurysms may cumulatively increase the risk of SAH (36). The PHASES score can calculate the rupture risk of the largest aneurysm in MIAs. Currently, there is no specific rupture risk assessment model tailored for patients with MIAs. To develop the PHASES score, 8,382 cases with IAs were reviewed, but our study included a relatively small number of patients (35). Our model relies on computers due to its complexity. In contrast, the PHASES score requires only a rating scale with six parameters. Only participants from North America and Europe other than Finland, Japan, and Finland were included to develop the PHASES score, and Chinese patients were not included. Our study, unlike the former ones, collected and analyzed data from Chinese patients, which can increase the generalizability of the results to Chinese people. Possibly due to the limited sample size, this study did not find significant differences in sex, age, or hypertension between the ruptured and unruptured aneurysm groups. Due to the constraints of a limited sample size and the single-center nature of the study, no significant differences were observed in sex, age, or hypertension between the ruptured and unruptured aneurysm groups in this research. Additionally, factors such as Population and Earlier subarachnoid hemorrhage were not included in this study. On the other hand, an external validation study conducted by Feng *et al*. on the PHASES score for predicting the rupture of MIAs showed that this scoring system had a lower prediction ability for the rupture of MIAs, with an AUC of 0.57 for the largest IA (37). Our model had an AUC of 0.843, suggesting a relatively greater accuracy. Five-fold cross-validation experiments revealed that our model had satisfactory prediction ability with a fine accuracy of 0.797. Nevertheless, the model could not satisfactorily predict the ruptured aneurysms (TPR = 0.644), which might be related to the small sample size. The performance of any diagnostic method or system relies on the balance between its sensitivity and false-positive rate. Additionally, the lack of external validation, which is essential to confirm the model’s robustness, limits the generalizability of our findings.

This study included both unruptured and ruptured aneurysms, which imposes certain limitations on the assessment of rupture risk for unruptured aneurysms. The morphological changes in aneurysms after rupture may significantly impact the reliability of the data. Utilizing retrospective data from ruptured aneurysms could affect the accuracy of our assessment of rupture risk in unruptured intracranial aneurysms (38, 39). In clinical practice, it is indeed challenging to capture the state of an aneurysm prior to its rupture. Consequently, this model may be more applicable in clinical scenarios aimed at identifying the responsible aneurysm in patients with multiple aneurysms who have experienced subarachnoid hemorrhage.

## 5 Limitations

In this article, the model’s performance in distinguishing ruptured aneurysms is inferior to that for unruptured aneurysms. This may be attributed to several factors. The mechanisms underlying aneurysm rupture remain unclear, and the model only incorporates two-dimensional morphological parameters related to rupture, excluding three-dimensional morphological parameters and other risk factors that may be more closely associated with aneurysm rupture. Second, the rupture of an aneurysm can change its size and shape, potentially increasing other morphological parameters and neck diameter (38, 39). However, our data were based on post-rupture DSA, and pre-rupture imaging data were missing. Therefore, accurate measurement of aneurysm structure is not feasible, which may affect the results. Thirdly, the absence of external validation limits the generalizability of the model. Future studies should include external validation to confirm the model’s performance. Additionally, this study is based on limited single-center data, which may also contribute to this limitation. Incorporating more risk factors and conducting multicenter studies will be the focus of our next phase of research. More importantly, this model does not aim to replace the decision and clinical experience of neurosurgeons but helps them optimize treatment decisions, reduce unnecessary surgical interventions, lower surgical risks, and improve treatment outcomes.

## 6 Conclusion

Based on our results, irregularly shaped aneurysms, aneurysms located at parent artery bifurcations, and aneurysms with a larger AR are at a higher risk of rupture. Moreover, our RPM represents a significant step forward in rupture risk prediction for MIAs, offering a more tailored approach than existing tools like the PHASES score. However, its clinical applicability is currently limited by suboptimal performance for ruptured aneurysms and the lack of external validation. Future studies should focus on larger, prospective cohorts and external validation to refine the model and enhance its clinical utility.

## Data Availability

The datasets presented in this study can be found in online repositories. The names of the repository/repositories and accession number(s) can be found in the article/Supplementary material.
